# Supplementary material for: Large-volume focus control at 10 MHz refresh rate via fast line-scanning amplitude-encoded scattering-assisted holography
Source: Nat Commun. 2024 Apr 8;15:2926. doi: 10.1038/s41467-024-47009-w (PMC11001868; doi:10.1038/s41467-024-47009-w)
Supplement: Supplementary file 1 — Supplementary Information [file 41467_2024_47009_MOESM1_ESM.docx]

**Supplementary Information**

**Large-volume focus control at 10 MHz refresh rate
via fast line-scanning amplitude-encoded scattering-assisted holography**

Atsushi Shibukawa et al.

Supplementary Text 1. Optimal line beam projection system

Here, we discuss the implementation of an optimal line beam projection system to further improve the spatial DOF and the temporal DOF in the proposed 1D modulation technique (i.e., focal contrast and focal refresh rate in the FLASH technique). The active number of DMD pixels is one of the critical factors that determines the overall performance of the proposed technique. The larger the number of active columns $M$ and rows $N$, the higher the refresh rate and the focal contrast. It is therefore desirable to construct a line beam projection system that can scan a line beam as thin as a single DMD column across the entire DMD surface.

In our experiments, we used a standard 4x objective lens (PLN4X, Olympus) with an NA of 0.1 to project a scanning line beam onto the DMD plane. With the standard field number of 22, the magnification factor of 4 results in the maximum field of view (FOV) of 5.5 mm, which limits the number of active columns or rows to around 400 with a single pixel dimension of 13.7 μm. The reason for choosing this objective lens is to minimize the width of the line beam, and thereby, to minimize the transient time for a line beam traveling across neighboring columns (i.e., rise and fall time of focal on/off modulation in the FLASH technique).

The DMD used has 1024 × 768 pixels with a pixel size of 13.7 μm. Therefore, the objective lens should have a scanning field of view (FOV) of around 14 mm and a spatial resolution of less than 13.7 μm to use the entire DMD surface for FLASH focusing with the setting of $M_{\mathrm{col}}=1$ (i.e., the number of columns illuminated with a single line beam is 1). There are commercially available 1x objective lenses that meet those requirements, such as M Plan Apo 1x from Mitutoyo and 1x ICO from Navitar. Also, the F-theta lens could be a good candidate because it can meet the above requirements as well. As the proposed technique is based on the retro-reflecting configuration, a conventional non-telecentric f-theta lens would not serve the purpose. For example, the one from Ronar Smith (TSL-532-15-58) would be a telecentric F-theta lens that meets the requirement. Given that the f-theta lens is telecentric-type and the angular scanning speed is constant as in polygon scanning mirrors, it provides the additional benefit of a constant refresh rate of focal spot because the scanning speed of the line beam over DMD columns becomes proportional to the angular scanning speed.

Because of the well-known trade-off relation between FOV and spatial resolution, it becomes more challenging to simultaneously achieve a short transient time (i.e., a line beam that is sufficiently thinner than the width of the DMD column) and FOV as large as the whole DMD plane. Indeed, when using the above-mentioned alternative such as a 1x objective lens or telecentric F-theta lens that can entirely address the whole DMD plane, the width of the line beam becomes as thick as the DMD column, thereby leading to the longer transient time as compared with the case for our current setting. As the laser power during this long transient time is simply wasted, the large energy loss of the CW laser beam would be caused in our modulation technique. When setting $M_{\mathrm{col}}$ to higher than 1, this energy loss is even more pronounced as the transient time gets longer. Also, when a thick line beam is positioned at the junction of optimized columns, it results in the undesired mixing between the two output optical fields. One effective solution to minimize such effects is to use a pulsed laser (preferably, with a repetition rate higher than 1MHz) synchronized with a line-beam scanning unit instead of using a CW laser as in our work.

Supplementary Text 2. Alternative methods to address focal spots in a 3D space

For finding out wavefront solutions to scan focal spots across different target planes (i.e., different axial positions), we repeatedly measured transmission matrices (TMs) at corresponding target planes (i.e., measured one TM for each target plane). However, it has been known that, once the TM is characterized at a specific target plane, optimal input fields for 3D addressable focus can be deduced from the characterized TM, in turn enabling the 3D focus control over a certain 3D range^1,2^. Here, we discuss the two possible approaches – basis transformation-based^1,2^ and memory effect-based^3^ – for 3D addressable focusing without multiple TM measurements and explain the perspective of those approaches for the FLASH focusing technique.

Basis transformation-based approach

In principle, given that a TM is characterized for a large transversal area at a fixed target plane $z=z_{0}$ whose transversal coordinate is set as $(x, y)$, one may perform a basis transformation on the measured TM to computationally construct a new TM with the output plane $z=z'$ whose transversal coordinate is set as $(x', y')$. More specifically, the TM for the new plane $z=z'$ can be constructed via $\mathbf{K}_{z=z'}=\mathbf{T}\times\mathbf{K}_{z=z0}$ where $\mathbf{K}$ is the transmission matrix at each plane and $\mathbf{T}$ is the transformation matrix whose column encodes the discretized spherical wavefront information (i.e., $e^{\pm ikr}$ where $r=\sqrt{\left( x^{'}-x \right)^{2}+\left( y^{'}-y \right)^{2}+\left( z'-z_{0} \right)^{2}}$. Note that the amplitude variation in the spherical wavefront is omitted for simplicity.) from the plane $z=z_{0}$ to the plane $z=z'$. Once the new TM is properly constructed, one may use a column of the phase-conjugated matrix $\mathbf{K}_{z=z'}^{*}$ to focus at a specific transversal position at the plane $z=z'$. This process can be in effect considered as the reconstruction of an ideal converging or diverging wavefront at the plane of $z=z_{0}$ which is focused at the plane of $z=z'$.

To ensure the lossless transformation, the original TM needs to be characterized for a large area with a high spatial resolution. For instance, it requires characterizing an extremely large transmission matrix with the number of output modes of around 10^8^ to address a micrometer-sized focus over a millimeter-scale volume as in our study. This process takes a prohibitively long time for a conventional scattering medium so that we opted to measure the transmission matrices multiple times in our study. It should be noted that the “randomness” of the TM can be specifically designed with a disorder-engineered metasurface platform^1^. We anticipate that such approaches can be incorporated with the proposed FLASH technique to get rid of the needs for repetitive TM measurements.

Memory effect-based approach

As an alternative, given that a scattering medium presents a sufficiently large memory effect range, one may superpose a spherical wavefront directly on a phase-conjugated field of the measured TM (i.e., a column of $\mathbf{K}_{z=z0}^{*}$) whose transversal coordinate is set as $(x, y)$. Specifically, one may take a column of $\mathbf{K}_{z=z0}^{*}$ corresponded to a desired focus at $(x_{f}, y_{f})$, then multiply discretized spherical wavefront $e^{\pm ikr}$ (where $r=\sqrt{\left( x_{f}-x \right)^{2}+\left( y_{f}-y \right)^{2}+\left( z_{f}-z_{0} \right)^{2}}$) to axially translate the focus to the plane of $z=z_{f}$. The key difference to the basis transformation strategy is that this strategy directly adds the spherical wavefront to the input wavefront and aims to utilize the intrinsic correlation within a transmission matrix. Therefore, the transmission matrix does not have to be characterized for the large number of output modes.

This correlation effect, often called short-range correlation, is formally defined in between transmission matrix elements for certain input and output wavevectors and only holds within a certain angular range which is inversely proportional to the thickness of a scattering medium *L*, more specifically, $\Delta\theta_{\mathrm{range}}\sim\lambda/2\pi L$. In practice, the opal diffusing glass with a near Lambertian scattering profile used in the experiment presented $\Delta\theta_{\mathrm{range}}<0.1^{\circ}$ as shown in Supplementary Figure 6, which is too narrow for addressing a millimeter-sized volume with a micrometer-sized focal spot. Moreover, due to our 1D wavefront modulation scheme, this strategy is only applicable to impose a 1D zone-plate-like pattern along a single direction like a tunable cylindrical lens.

Supplementary Text 3. Intensity fluctuation of focal spots from a stochastic property of transmission matrix

The intensity fluctuation of focal spots shown in Figs. 3e, 4c, and 5d can be partially attributed to intrinsic statistical fluctuation in the transmission matrix of scattering media. Here, we provide a detailed discussion about the statistical fluctuation and present the fluctuation level estimated from numerical simulation.

The relation between input modes (i.e., DMD pixels) and output modes (i.e., independent speckle granules) can be described based on the transmission matrix formalism: $E_{m}^{\mathrm{out}}=\sum_{n} t_{m,n}E_{n}^{\mathrm{in}}$ where $E_{m}^{\mathrm{out}}$ and $E_{n}^{\mathrm{in}}$ are complex fields of the $m$th output mode and the $n$th input mode. $t_{m,n}$ is the transmission matrix element that relates the $m$th output mode with the $n$th input mode. The matrix elements, $t_{mn}$, are statistically independent and follow a complex Gaussian distribution.

The binary phase conjugation process used in our experiments is to selectively turn on the input modes (DMD pixels) that additively contribute to the intensity of a target output mode, $\left| E_{\mathrm{target}}^{\mathrm{out}} \right|^{2}$ (e.g., to choose input modes with $\left| \arg\left( t_{target,n} \right) \right|<\pi/2$). Therefore, roughly half of the input modes are set on and another half are set off. When “ensemble averaged”, this process results in the peak intensity, $\left\langle\left| E_{\mathrm{target}}^{\mathrm{out}} \right|^{2} \right\rangle$, $N/(2\pi)$ times enhanced compared to the background intensity, $\left\langle\left| E_{\mathrm{background}}^{\mathrm{out}} \right|^{2} \right\rangle$, where $N$ is the total number of controlled input modes.

In our experiments, we corrected wavefront distortion for different DMD columns to achieve an optimal peak intensity (as shown in Supplementary Fig. 7). Therefore, the binarized phase conjugation process is modified to the process of turning the input modes with $\left| \arg\left[ t_{target,n}\times e^{i\Delta\varphi_{n,k}} \right] \right|<\pi/2$ into the “ON”-state where $\Delta\varphi_{n,k}$ is the column-dependent distortion-correction pattern (i.e., correction pattern for the $k$th column). Therefore, although we optimized the wavefront for the fixed positions in Figs. 4e, 4c, and 5d, the 1D binarized patterns vary substantially for different DMD columns. This effect is evidenced in Supplementary Fig. 7 which shows the similar background patterns without wavefront correction (i.e., when the same 1D binary pattern is repeated) and the varying background patterns with wavefront correction.

Due to this aspect, each realization of phase conjugation process for different DMD columns is subject to statistical fluctuation from the intrinsic stochastic property of the transmission matrix (i.e., statistical fluctuations in adding different sets of random phasors). The same fundamental stochastic noise should be observable when focal spots are created at different positions. Setting the peak intensity as $I_{\mathrm{target}}=\left| E_{\mathrm{target}}^{\mathrm{out}} \right|^{2}$, the fluctuation level can be characterized as the ratio of the standard deviation $\sigma_{I}$ to the average value $\mu_{I}$ of $I_{\mathrm{target}}$. With the numerical simulation of repeated phase conjugation process through different transmission elements, we found that this fluctuation level is scaled with $\sqrt{N}$, which is consistent with the central limit theorem. The simulated fluctuation levels for phase-only and binary-amplitude modulation for different value of $N$ were plotted with fitted curves in the Supplementary Figure 9. For our case of binary-amplitude modulation with $N=352$, the fluctuation level is estimated to 15 %.

Supplementary Text 4. Potential applications of FLASH technique

In our proposed scheme, the focal contrast (henceforth, peak-to-background ratio will be used as the terminology for focal contrast) is compromised to boost the speed of modulation, resulting in the experimental peak-to-background ratio (PBR) of around 40 at the setting of $M_{\mathrm{col}}=1$. This limited PBR would be the most critical point when considering the practical applicability of the proposed technique. The non-zero background is the general feature that is commonly observed in wavefront shaping techniques for scattering media (i.e., interferometric focusing) due to the non-unitarity of associated transmission matrices. The usability of interferometric focusing schemes has been explored extensively since early pioneering works based on wavefront shaping and optical transmission matrix^1,3–7^. Here, based on the demonstrations from those studies, we discuss the potential usability of the FLASH technique in two aspects – non-imaging^4–6^ and imaging applications^1,3,7^.

Non-imaging Applications

At the current stage, we consider that the FLASH focusing technique would be best suited for non-imaging application areas in which some nonlinear processes that can reduce the effect of speckle backgrounds are involved and a specific region within a large volume is important at a time (i.e., not all the positions of the specimen are simultaneously addressed). Many practical demonstrations have already been reported based on scattering-aided interferometric focusing schemes with surrounding background fluctuations. For instance, deep tissue optogenetic stimulation of a targeted single neuron has been demonstrated with a PBR as high as ~2.^6^ In this study, although the PBR was extremely low, the interferometric focusing could be practically used to selectively stimulate the targeted neuron based on the nonlinear characteristic of optogenetic proteins (i.e., switching behavior over a light intensity threshold). Similarly, optical trapping through a scattering medium^5^ and tissue ablation^4^ have also been successfully demonstrated.

Imaging Applications

FLASH focusing technique, like other scattering-aided interferometric focusing methods, accompanies speckle backgrounds (i.e., speckle granules around the focal spot), which we consider is the key disadvantage in comparison to conventional varifocal lenses. The key point to make the FLASH technique practically useful for imaging applications is therefore to incorporate a mechanism that can minimize the effect of those speckle backgrounds. Considering that the imaging system is comprised of an illumination path and a detection path, the interferometric focusing scheme with the FLASH technique only serves the illumination path. Here, we present three potential ways to implement detection paths in the scanning imaging system that can effectively suppress the effect of the low PBR in the FLASH focusing-based imaging applications.

**1) Confocal or quasi-confocal configuration in transmission mode:** One may configure the optical sensors in confocal or quasi-confocal positions with a limited collection area to suppress the signal from background regions.^1,3^ In this strategy, the image contrast is highly dependent on the PBR of the focal spot and the size of the collection area (i.e., the pinhole area in confocal microscopy), which will be discussed in detail in the Supplementary Text 5 that describes the additional experimental procedure and results in the Supplementary Figure 12.

**2) Wavefront shaping for epi-detection scheme:** Because of scattering media, conventional epi-detection schemes (i.e., scan and descan with same scanning mirrors) cannot be directly applied in interferometric focusing schemes. However, as demonstrated in the previous work^7^, a detection path can also be implemented with wavefront shaping techniques. In this work, it has been shown that the narrow-band signal of fluorescence emission from a focal spot can be selectively detected via the phase conjugation process without the effect of the backgrounds. This configuration would provide the confocal image with the contrast of the PBR for excitation multiplied by the PBR for detection.

**3) Multiphoton excitation:** We anticipate that multiphoton fluorescence imaging is one of the promising ways to implement an imaging system while circumventing the effect of the low PBR. Like a conventional two-photon microscope, we expect that one may retrieve a high-contrast image with a photomultiplier tube that collects a total amount of fluorescence as demonstrated in the recent work^8^*.* We note that, in this study, the PBR of the focal spot was around 300 (i.e., the effective PBR for the fluorescence signal was ~ 10^5^) which is achievable by adjusting $M_{\mathrm{col}}$ in our setup or using a commercial DMD with a high resolution of 1920 × 1080.

Supplementary Text 5. Experimental demonstration of scanning fluorescence imaging with FLASH technique

We performed scanning imaging of fluorescent microspheres and fluorescence-stained biological samples with the FLASH focusing technique based on the confocal or quasi-confocal configuration in transmission mode as described in Supplementary Text 4.

**Experimental setting and image acquisition process**

As shown in Supplementary Fig. 12a, a focal spot was raster-scanned on a sample plane positioned 3mm behind the scattering medium, and the excited fluorescence from a sample passing through a longpass filter (FELH0550, Thorlabs) was captured on a CMOS camera in a microscope setup. As illustrated in Supplementary Fig. 12b, the fluorescence excited at each scan position over a raster-scan path was captured by the camera and the integration of the fluorescence intensities over a collection area of a diameter $\varphi_{\mathrm{pin}}$ (green circle in Supplementary Fig. 12b) on the captured image was given as the value of the corresponding pixel in the scanning image under construction (green square in Supplementary Fig. 12b). After repeating this procedure for all scan positions along the entire raster-scan path, the scanning fluorescence image was constructed. This imaging scheme is similar to that of conventional confocal microscopy, except that backgrounds from out-of-focus positions are digitally removed by setting up a pinhole-like collection area in the captured image. When raster-scanning the focal spot, the 1D resonant scanner was fixed and a central DMD column was updated to circumvent the experimental complexity of synchronizing the 1D scanner and the camera.

Varying collection area and its practical implication for imaging applications

We have demonstrated the capabilities of the MHz scanning speed and the addressability to millimeter-scale volume using the FLASH technique. One potential configuration in the detection path that can take advantage of those capabilities in imaging application would be to use a low-magnification objective lens and a high-speed camera with a MHz frame rate. As the practical example, our previous study based on the scattering-assisted focusing scheme^1^ used a standard 4x objective lens to demonstrate scanning fluorescence imaging with the effective NA of 0.5 over a wide field-of-view (FOV) of 8 mm diameter that is inaccessible in conventional microscope. Instead of obtaining a beneficial wide-FOV, the use of the low magnification objective in the detection path results in the spatial resolution of the detection system (i.e., the detection PSF) that is sufficiently smaller than the interferometric diffraction-limited spot (i.e., the illumination PSF), meaning that the integration of fluorescence intensities over the collection area whose diameter is set to the size of the detection PSF includes not only the signal from the focal spot but also the noises from surrounding aggregated backgrounds.

In the following scanning fluorescence imaging experiment, we varied the diameter of the collection area (i.e., varying the diameter of the pinhole in a quasi-confocal configuration) to see the effect of aggregated backgrounds and also to find out what magnification of objective lens can be chosen under the present focal contrast of the FLASH technique. Specifically, the diameter of the collection area, *φ*_pin_, was set to 0.36 µm, 0.65 µm, 1.08 µm, 2.49 µm which corresponds to the FWHM of the diffraction-limited spot of off-the-shelf objective lenses with magnifications of 60x, 20x, 10x, and 4x. These values are based on standard objective lenses provided by Olympus (MPlanApo N 60x, UPlanFL N 20x, UPlanFL N 10x, UPlanFL N 4x).

Experimental results

For imaging fluorescent microspheres, with a spot size of 0.57 μm and a step size of 0.09 μm, the raster-scanning was performed over 11 × 11 μm^2^, resulting in 120 × 120 illumination spots (i.e., image resolution). For the biological specimens, the raster-scanning was performed over 16 × 16 μm^2^ with a step size of 0.18 μm, resulting in 90 × 90 image resolution. We used sparse sample and contiguous biological sample - diluted fluorescence microspheres (F13082, Invitrogen) and HeLa cells stained with a high-affinity F-actin probe conjugated to rhodamine fluorescence dye (ActinRed™ 555 ReadyProbes™ Reagent, Invitrogen).

The imaging results are shown in Supplementary Figs. 12c-l along with the wide-field fluorescence images excited by a collimated laser beam illumination. In the tight confocal configuration of *φ*_pin_ = 0.36 µm corresponding to the FWHM of the 60x objective, the results presented well-resolved images whose structural details are well-matched with the wide-field imaging results. In fact, as the confocal configuration provides depth-sectioning capability, the imaging results presented finer structural details compared to the wide-field results. We note that, due to the low fluorescence level, the stochastic camera noise slightly degrades the imaging results for the biological sample. Even in the case of *φ*_pin_ = 1.1 µm corresponding to the FWHM of the 10x objective where the collection area is around 4 times (= (1.08/0.57)^2^) bigger than the focal spot size, the image qualities were quite robust against the effect of speckle backgrounds. Those imaging results indicate that the combined use of the FLASH illumination and the detection system with the low magnification objective could result in a usable imaging contrast for the wide-FOV (e.g., 3 mm × 3mm FOV for 10x objective) in the presence of the effect of the aggregated backgrounds. In summary, we anticipate that the combined use of a low-NA objective lens and an ultra-high-speed camera with a MHz frame rate (e.g., HPV-X2, 10MHz frame rate at full resolution, Shimadzu) would be a practical detection system in the transmission geometry where the FLASH technique will be useful for imaging applications.

Lastly, when the collection area becomes 20 times larger than the spot size (as shown in the case of *φ*_pin_ = 2.5 µm), the aggregated backgrounds obscured the signal from the peak so that the structural details were missed. Compared to the sparse microsphere sample, this effect is more pronounced in the case of contiguous biological sample.


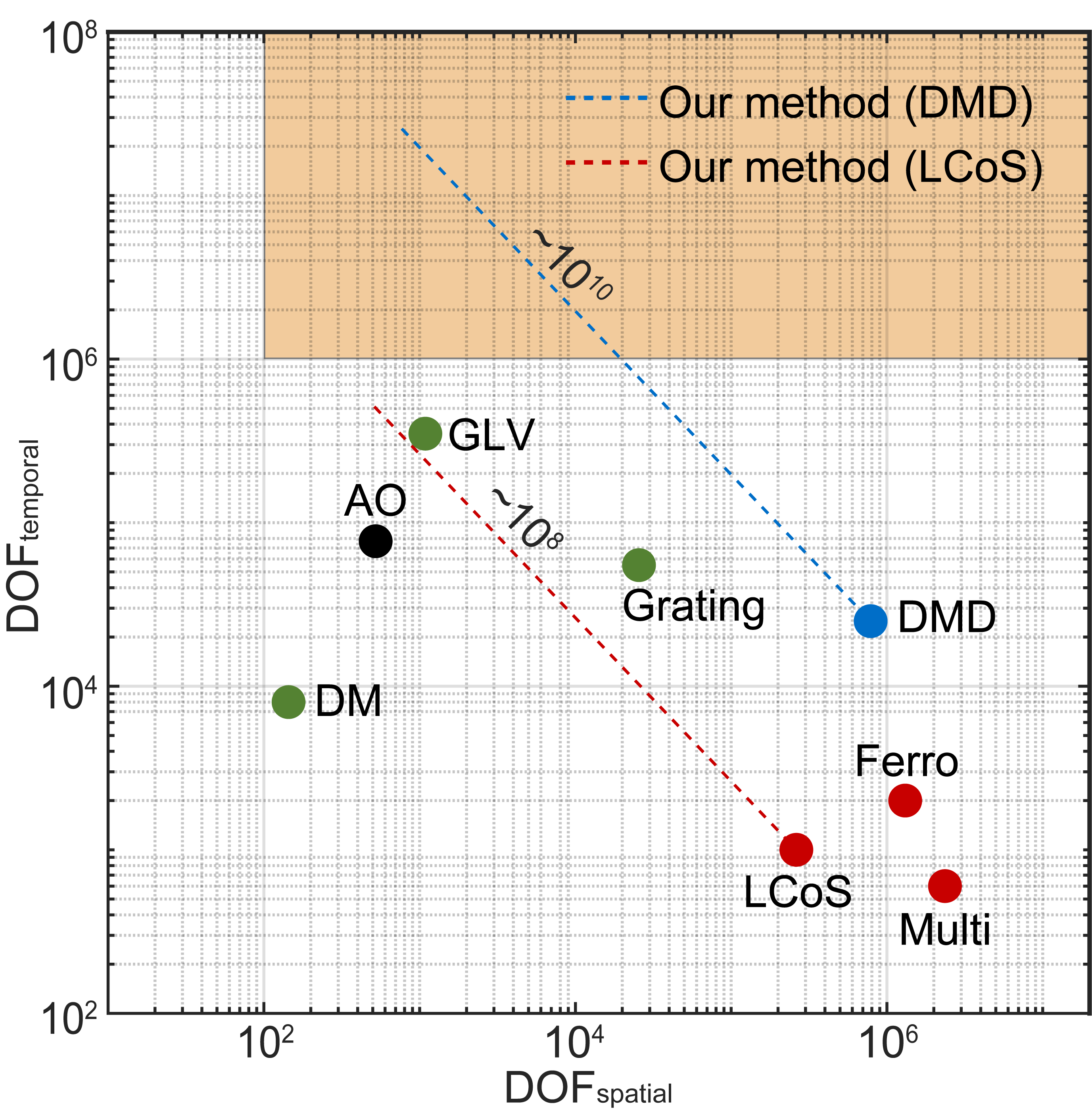
Supplementary Figure 1

**Supplementary Figure 1 |** Comparison of our wavefront modulator and other existing modulators in terms of $\mathrm{DOF}_{\mathrm{spatial}}$ and $\mathrm{DOF}_{\mathrm{temporal}}$. Phase-only LCoS (LCoS)^9^, ferroelectric LCoS (Ferro)^10^, multiple-SLM (Multi)^11^, DM^12^, GLV^13^, grating phase shifter (Grating)^11^, DMD^14,15^, and AO^16^ types are presented as existing SLMs. Note that EO-based SLMs^17–19^ are omitted from this comparison as its $\mathrm{DOF}_{\mathrm{spatial}}$ of smaller than 10^2^ is insufficient for 3D focus control. Dotted red and blue lines indicate ideal addressable DOFs using our methods based on DMD and LCoS, respectively. Filled orange area denotes the range where $\mathrm{DOF}_{\mathrm{temporal}}$ > 10^6^ and $\mathrm{DOF}_{\mathrm{spatial}}$ > 10^2^.


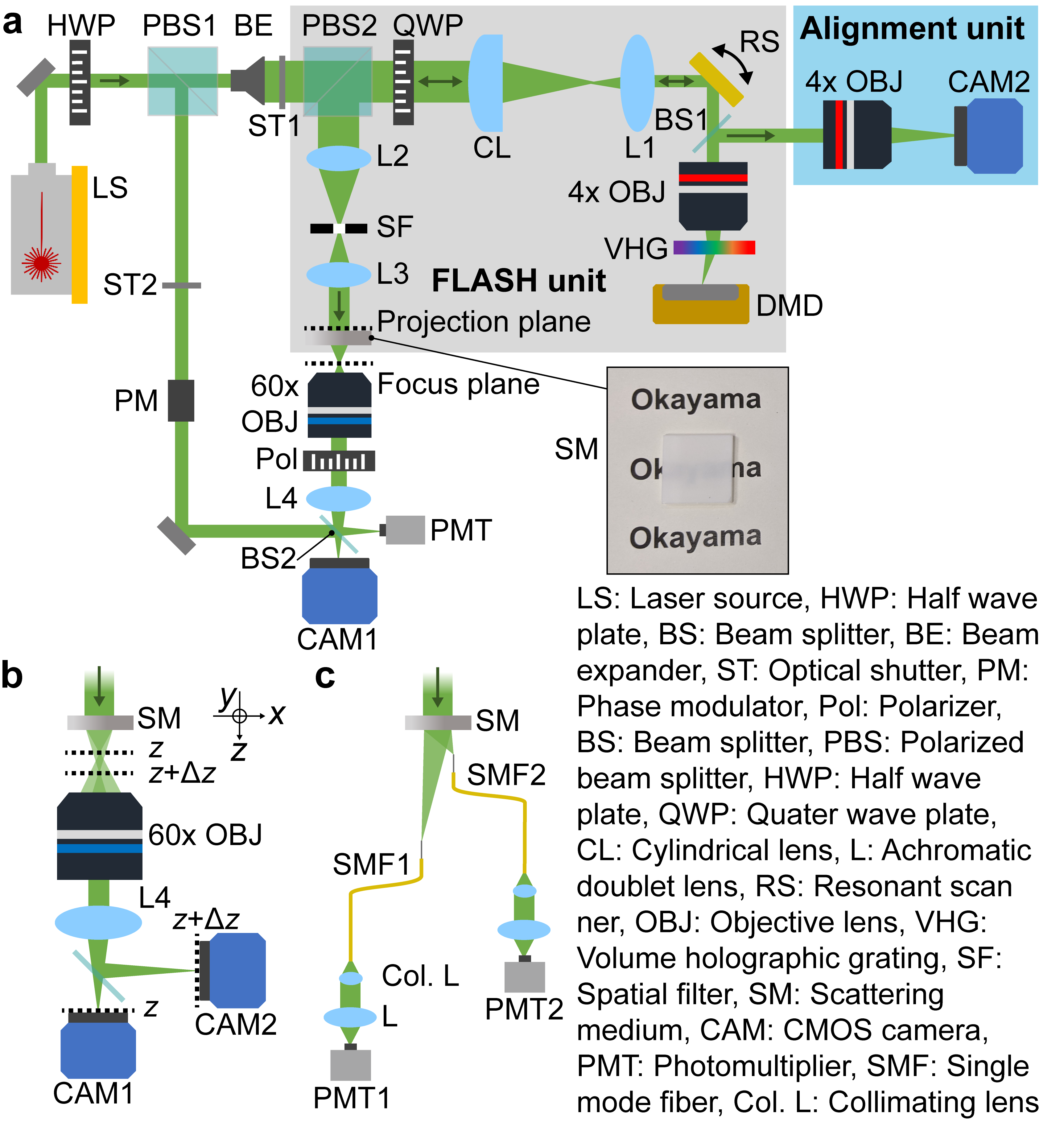
Supplementary Figure 2

**Supplementary Figure 2 |** Detailed optical setup for the demonstration of full capability of FLASH focusing technique. **a**, Entire optical setup that includes FLASH unit and alignment unit indicated by filled gray and blue areas, respectively. Focal lengths of lenses used in the setup are 100 mm (CL), 50 mm (L1), 150 mm (L2), 100 mm (L3), 150 mm (L4), respectively. **b**, Optical setup for 3D random-access focus control using two cameras (CAM1 and CAM2) that are positioned to image the target plane of *z* = 3 mm and *z* = 3.2 mm, respectively. **c**, Optical setup for large volume, frequency-multiplexed modulation of distant focal spots using two single mode fibers (SM400, Thorlabs). Focal spots behind the scattering medium are coupled into SMF1 and SMF2 and detected by two photomultiplier tubes (PMT1 and PMT2) through relay optics with collimating lenses (Thorlabs, F240APC-532, CL) and doublet lenses (L) with a focal length of 50 mm.


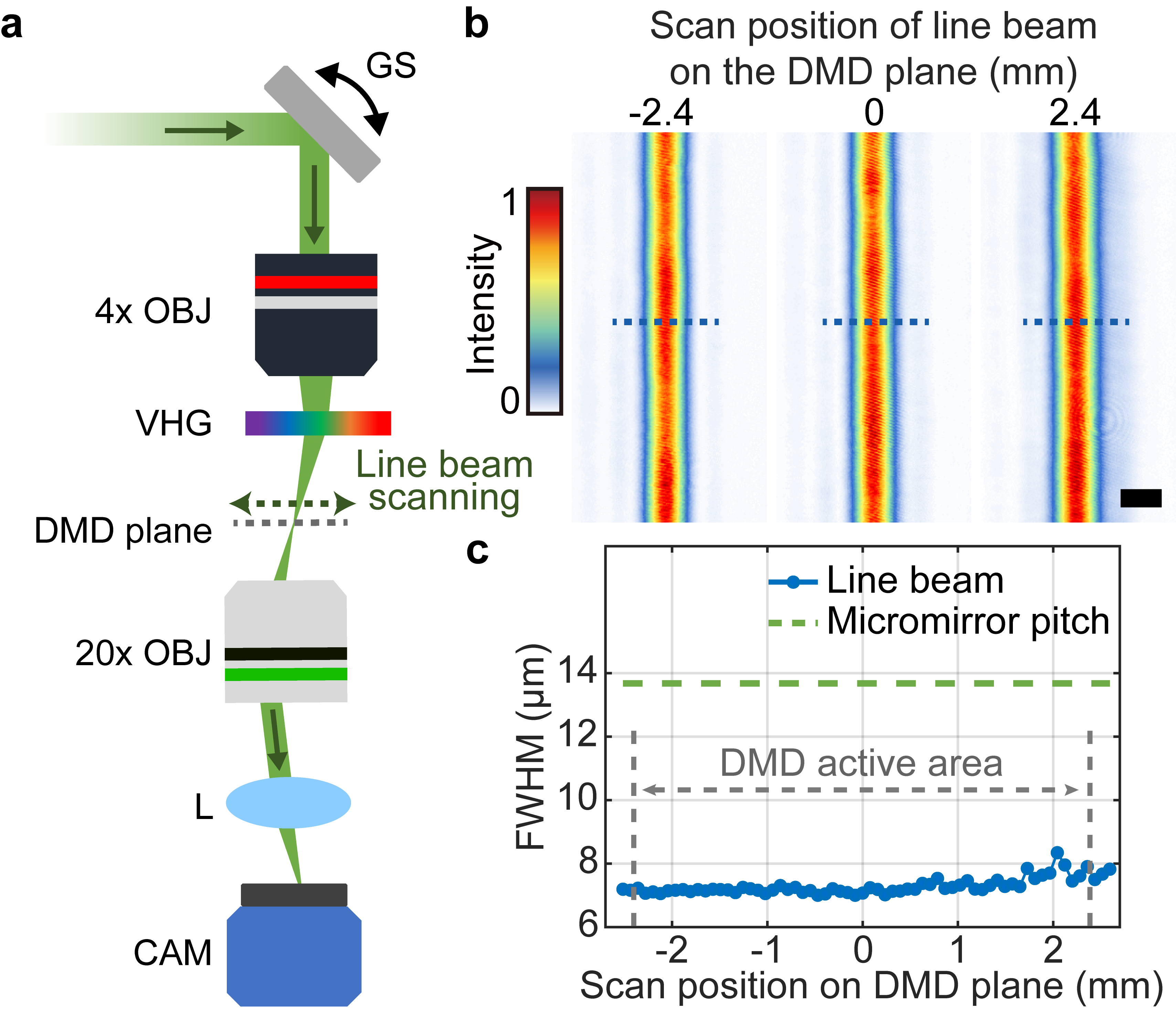
Supplementary Figure 3

**Supplementary Figure 3 |** Validation of the width of a scanning line beam through a volume holographic grating (VHG) over the DMD plane in the setting of $M_{\mathrm{col}}=1$. **a**, Optical setup for measuring the width of the line beam on a DMD plane. A microscopic setup composed of 20x objective lens (OBJ), tube lens (L), and a camera (CAM) was constructed to observe 2D intensity profile of the line beam on the DMD plane. To measure the width of the line beam in a stationary state at any specific positions, the resonant scanner was replaced with a Galvano scanner (GS). **b**, 2D profiles of the line beams at representative positions in the scan direction. Scale bar: 10 μm. **c**, FWHM of 1D profile along the blue dotted lines in **b**. The micromirror pitch (pixel size) of the DMD is indicated by the dashed green line. Within the entire width (~ 4.6 mm) of all 340 DMD columns denoted as ‘DMD active area’, the FWHM was consistently smaller than the micromirror pitch of 13.7 μm.


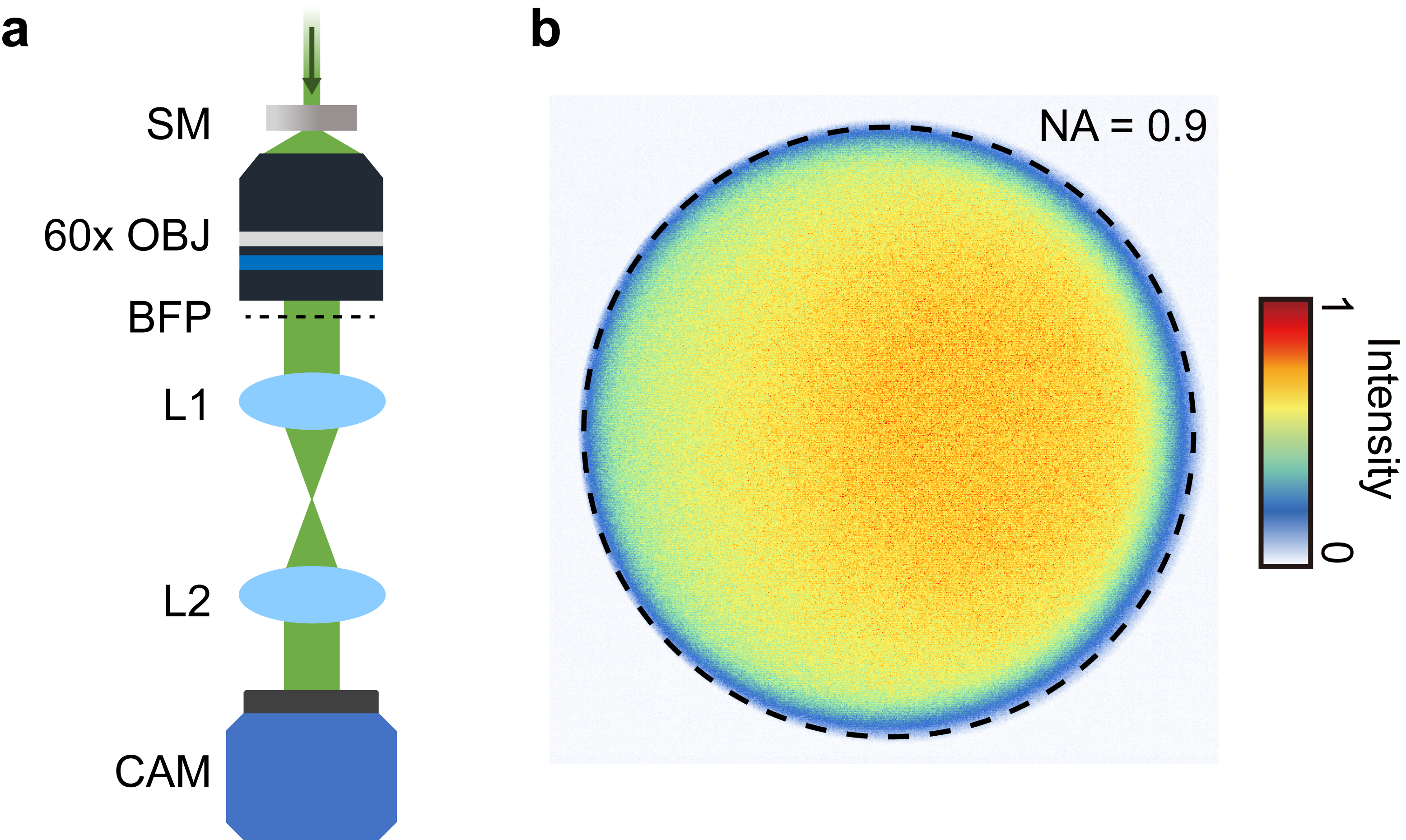
Supplementary Figure 4

**Supplementary Figure 4 |** Observation of a uniform and highly scattering profile of our scattering medium. **a**, Optical setup for measuring the scattering profile of scattering medium (SM). The pencil laser beam with a diameter of 0.5 mm was injected onto the medium. The intensity of the scattered beam on the back focal plane (BFP) of the 60x objective (60x OBJ) was imaged on the camera (CAM) through achromatic lenses of L1 and L2. The focal lengths of L1 and L2 are 150 mm and 150 mm, respectively. **b**, 2D scattering intensity profile of the scattering medium detected by the 60x OBJ with the numerical aperture (NA) of 0.9.


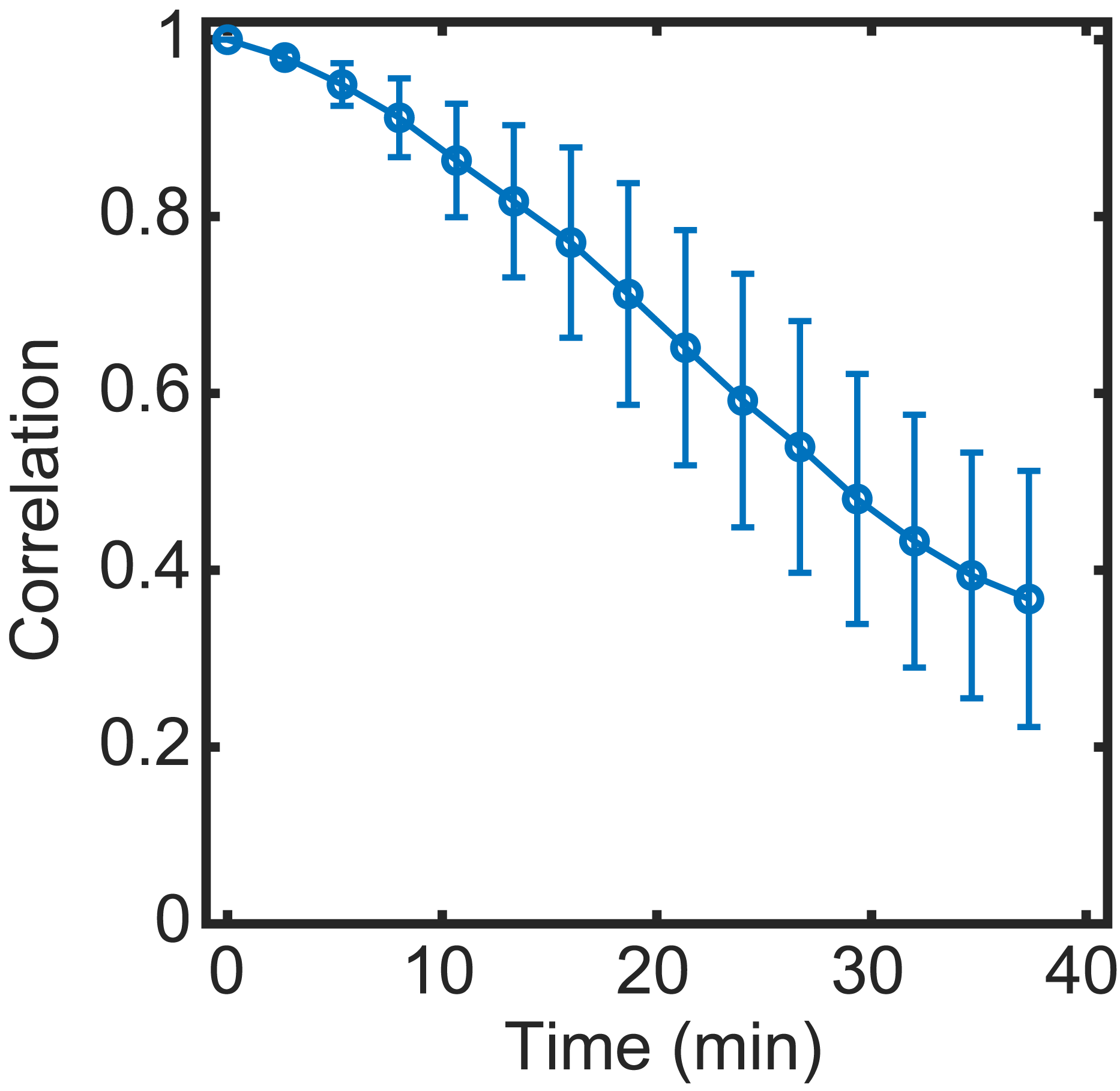
Supplementary Figure 5

**Supplementary Figure 5 |** Correlation coefficient between the first speckle pattern and subsequent speckle patterns as a function of time for an opal diffusing glass. A laser beam with a beam diameter of 4 mm was injected into the opal diffusing glass which was clamped with a filter mount (Thorlabs, DH1/M). Then, speckled patterns behind the opal glass at the target plane of *z* = 2 mm were captured through a microscopic setup consisting of a 60x objective lens and a tube lens to calculate correlation coefficients. The error bar shows the standard deviation of five measurements.


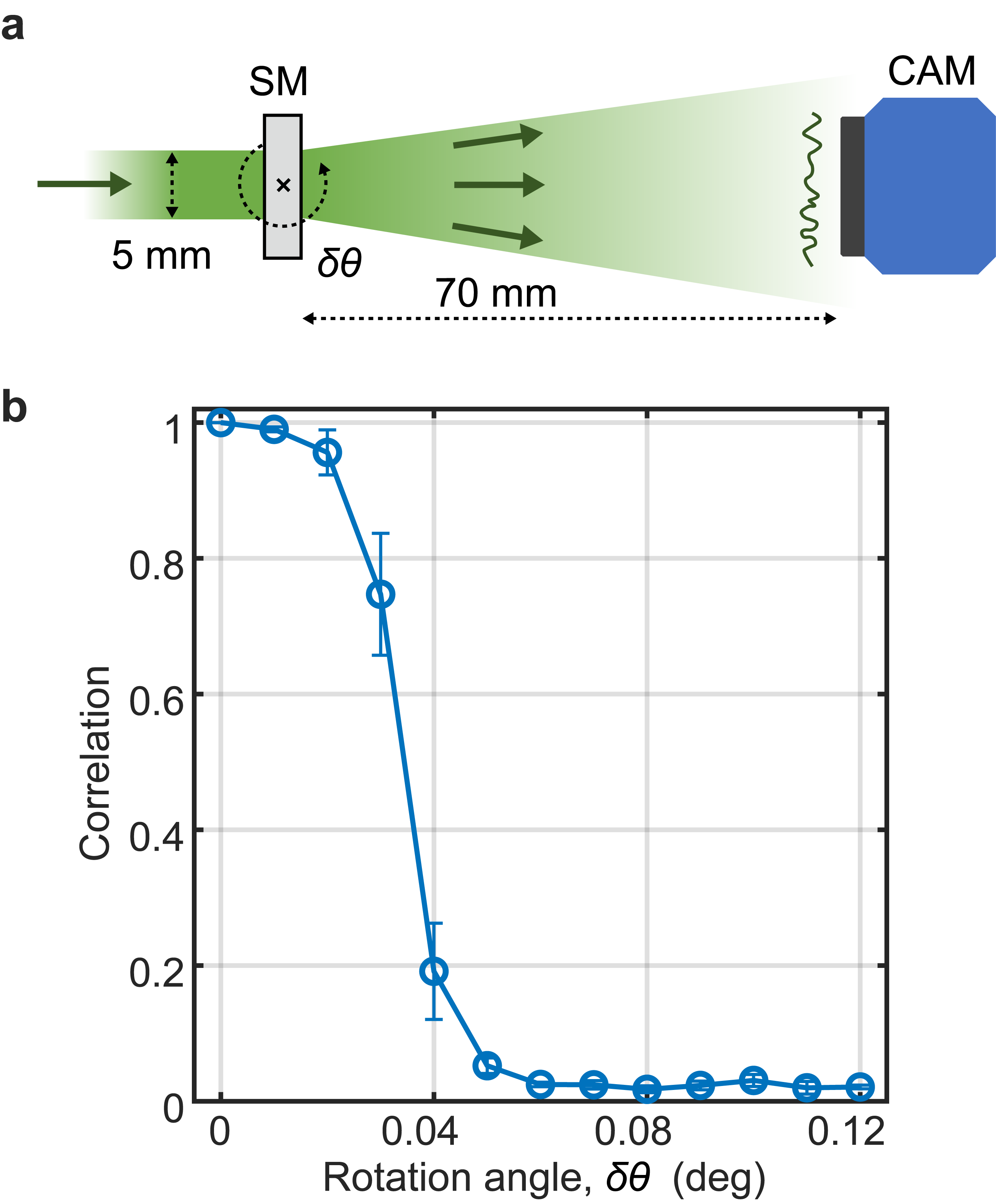
Supplementary Figure 6

**Supplementary Figure 6 |** Measurement of angular range of memory effect. **a**, Schematic of the optical setup to measure the angular correlation range of an opaque diffusing glass as a scattering medium (SM). A laser beam with a diameter of 5 mm was illuminated into the SM, and the speckled pattern was captured by a camera (CAM) at the plane 70 mm behind the SM. A 5 mm-diameter mask was placed on the medium so that the medium can be rotated without changing its illuminated area. **b**, Measured memory effect range for the SM. Correlation coefficient between the first speckle pattern at the rotation angle of $\delta\theta=0^{\circ}$ and the subsequent speckle pattern at each $\delta\theta$ was computed. Error bars indicate the standard deviation of five measurements.


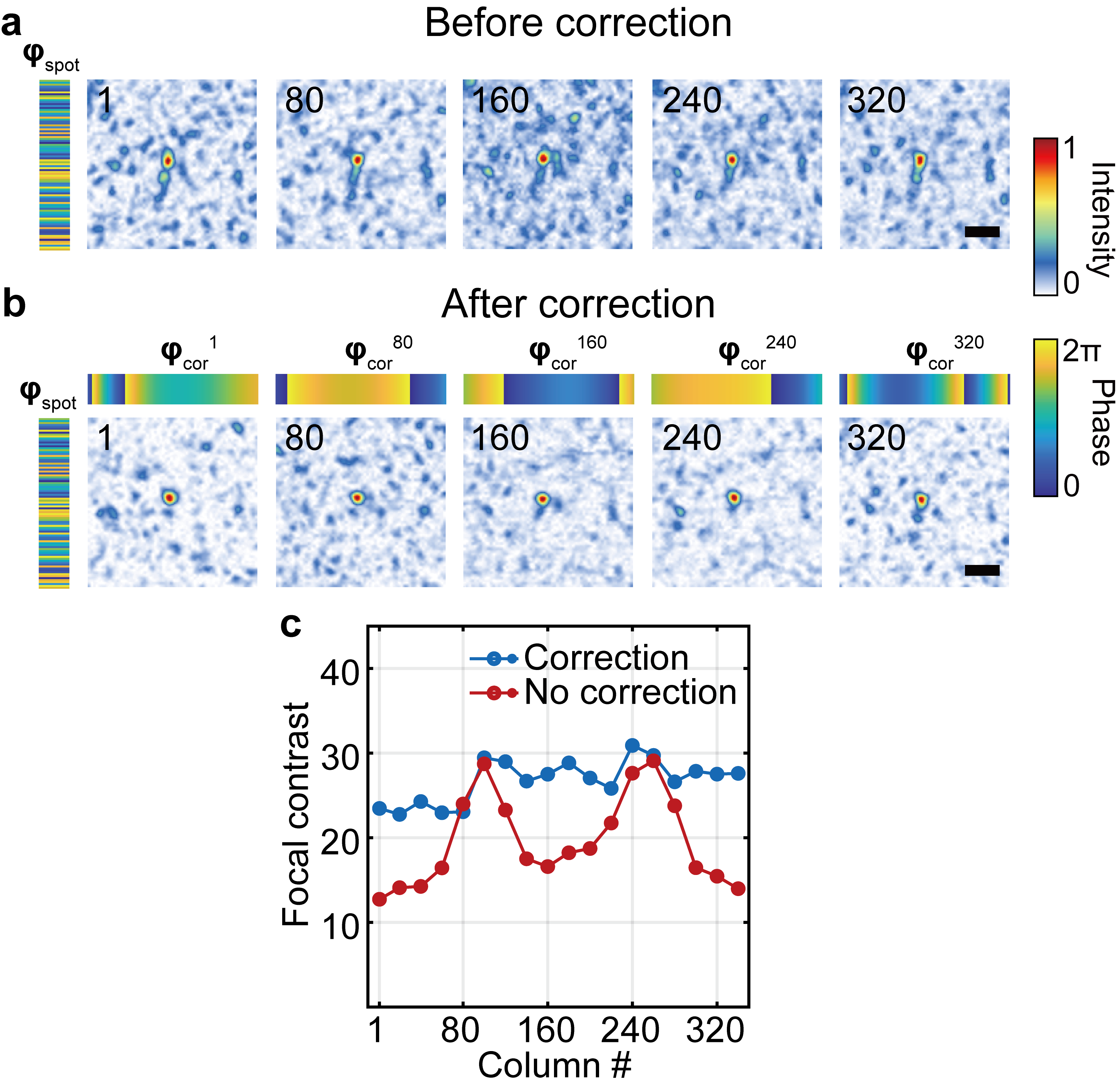
Supplementary Figure 7

**Supplementary Figure 7 |** Correction of column-dependent wavefront distortions using a few Zernike modes. **a**, Focal spots reconstructed from representative DMD columns without our correction method. Left panel shows the phase pattern $\boldsymbol{\varphi}_{\mathrm{spot}}$ calculated from the measured transmission matrix of the scattering medium. The binarized amplitude pattern converted from this phase pattern was individually displayed onto representative DMD columns. Scale bar: 1 μm. **b**, Focal spots reconstructed with our correction method. Upper panels show correction phase patterns $\boldsymbol{\varphi}_{\mathrm{cor}}$ for each representative column. Scale bar: 1 μm. **c**, Contrast of focal spots for different DMD column with and without the wavefront correction method.


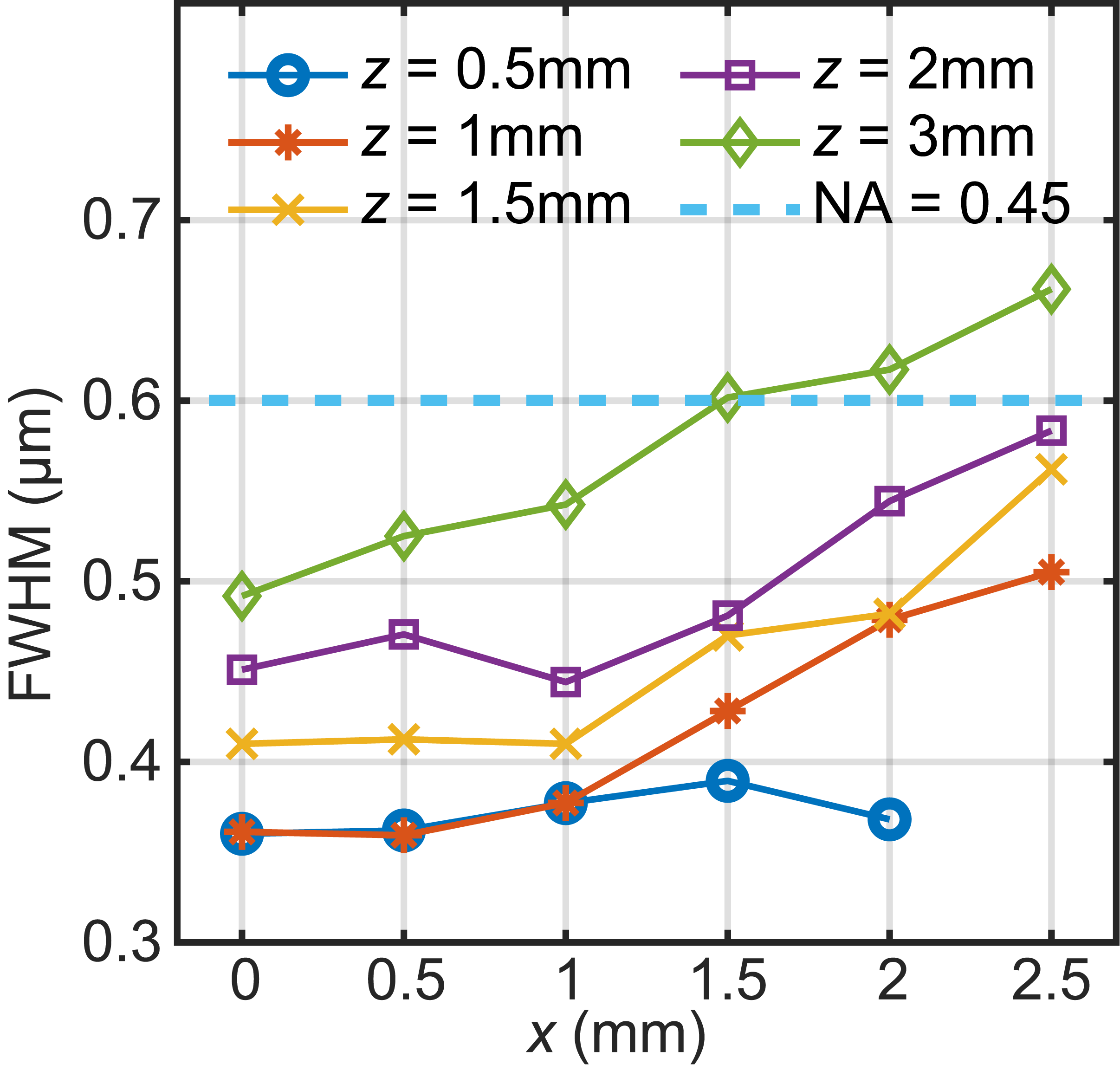
Supplementary Figure 8

**Supplementary Figure 8 |** Measured FWHM of foci for different *x* position behind a scattering medium at the target planes of *z* = 0.5mm, 1mm, 1.5mm, 2mm, and 3mm. The theoretical FWHM for diffraction-limited focal spot with NA of 0.45 at the wavelength of 532 nm is indicated by the dotted blue line for reference.


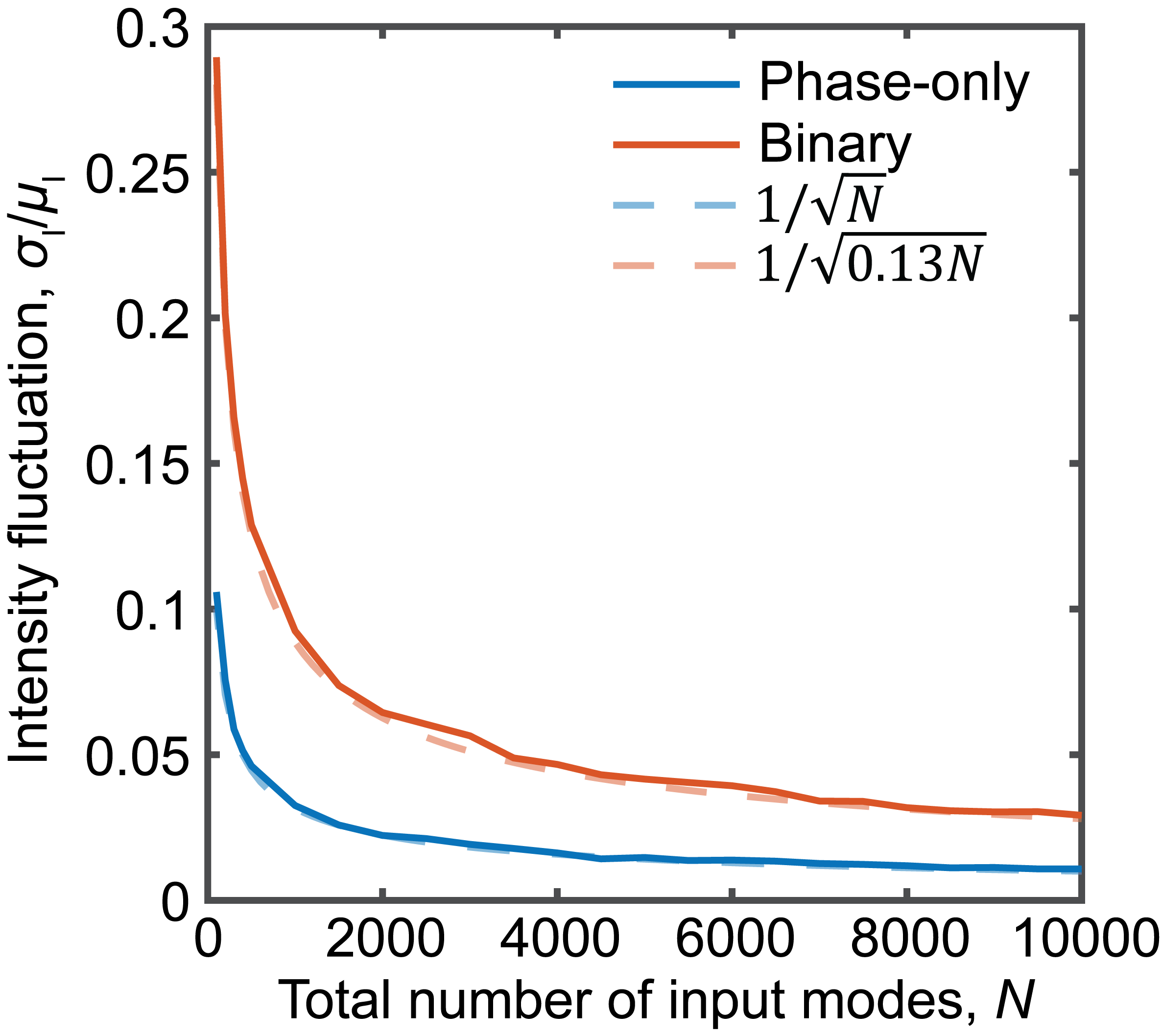
Supplementary Figure 9

**Supplementary Figure 9 |** Simulated intensity fluctuation of a focal spot for different total number of input modes $N$. Intensity fluctuation level was defined as the ratio of the standard deviation $\sigma_{I}$ to the mean value $\mu_{I}$ of the focal peak intensity. For each value of $N$, the simulation was repeated 1,000 times to get the statistically valid fluctuation level. The plots for phase conjugation reconstruction with phase-only and binary-amplitude modulations were fitted with $1/\sqrt{N}$ and $1/\sqrt{0.13N}$, respectively.


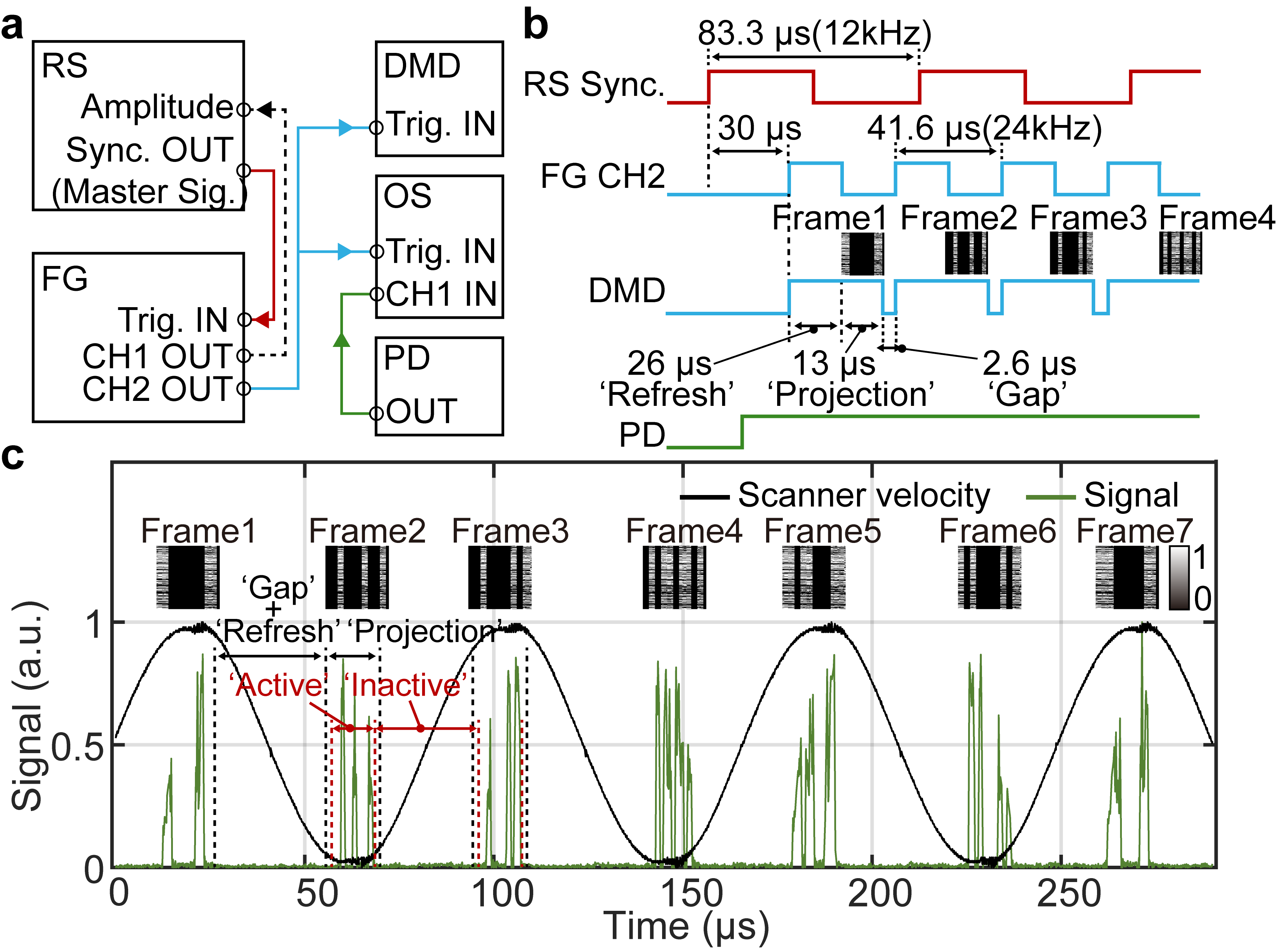
Supplementary Figure 10

**Supplementary Figure 10 |** Demonstration of continuous modulation capability in the proposed wavefront modulation technique. **a**, System control diagram. FG and OS denote a function generator and an oscilloscope, respectively. **b**, Electrical signal flow diagram. When the FG detects the first rising edge of the master synchronization signal (RS Sync) from the RS controller board, the CH2 of the FG outputs a pulse train of 24 kHz to trigger the refresh of DMD frames. Once the DMD board detect the first rising edge of pulsed signals from the FG, the DMD start refreshing an old frame during ‘Refresh’ time of 26 μs, followed by the mechanically stable ‘Projection’ time of a new frame (Frame 1) for the next 13 μs. Before the second rising edge from the FG is detected by the DMD, there is a ‘Gap’ time of 2.6 μs. **c**, Optical signals acquired with the PD for the on/off continuous modulation of focal spots. Inserted images indicate 2D binary patterns that are sequentially displayed on the DMD based on the pulsed trigger signal from the FG. ‘Active’ time and ‘Inactive’ time indicate the time for serial wavefront modulation using 340 columns and the time for the update of the DMD frame, respectively. The velocity signal from the RS controller board is plotted for reference in black line.


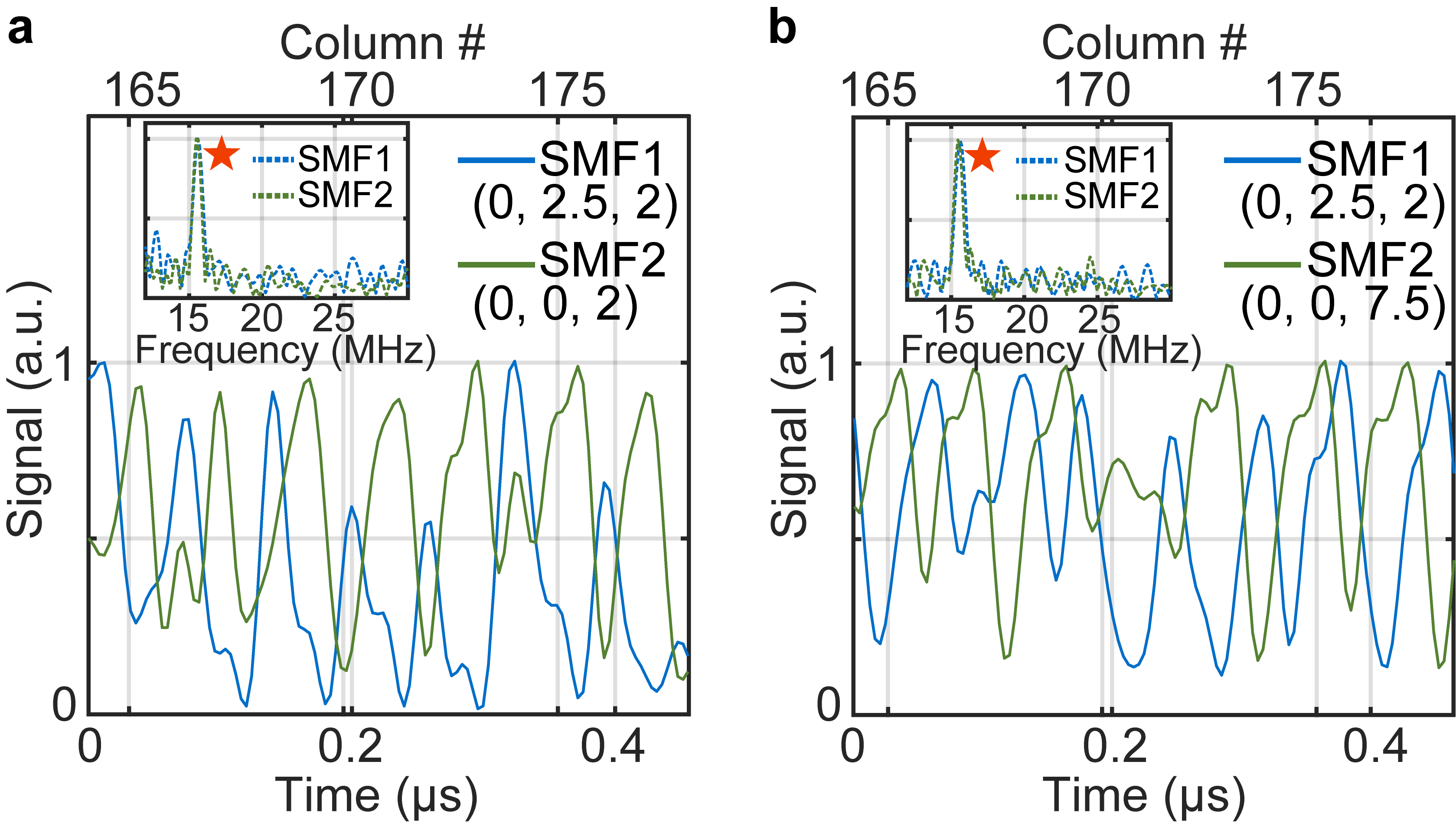
Supplementary Figure 11

**Supplementary Figure 11 |** Demonstration of ultrafast focus control over a large 3D volume using two distantly placed single-mode fibers. **a-b**, Optical signals for the on/off modulation of a focal spot over proximal ends of fibers. Two fibers were positioned at (*x*, *y*, *z*) = (0, 2.5, 2) mm and (0, 0, 2) mm in **a** and at (*x*, *y*, *z*) = (0, 2.5, 2) mm and (0, 0, 7.5) mm in **b**, respectively. Inset plots show frequency spectra for the optical signals in **a** and **b**. Orange stars indicate peaks in frequency spectra.


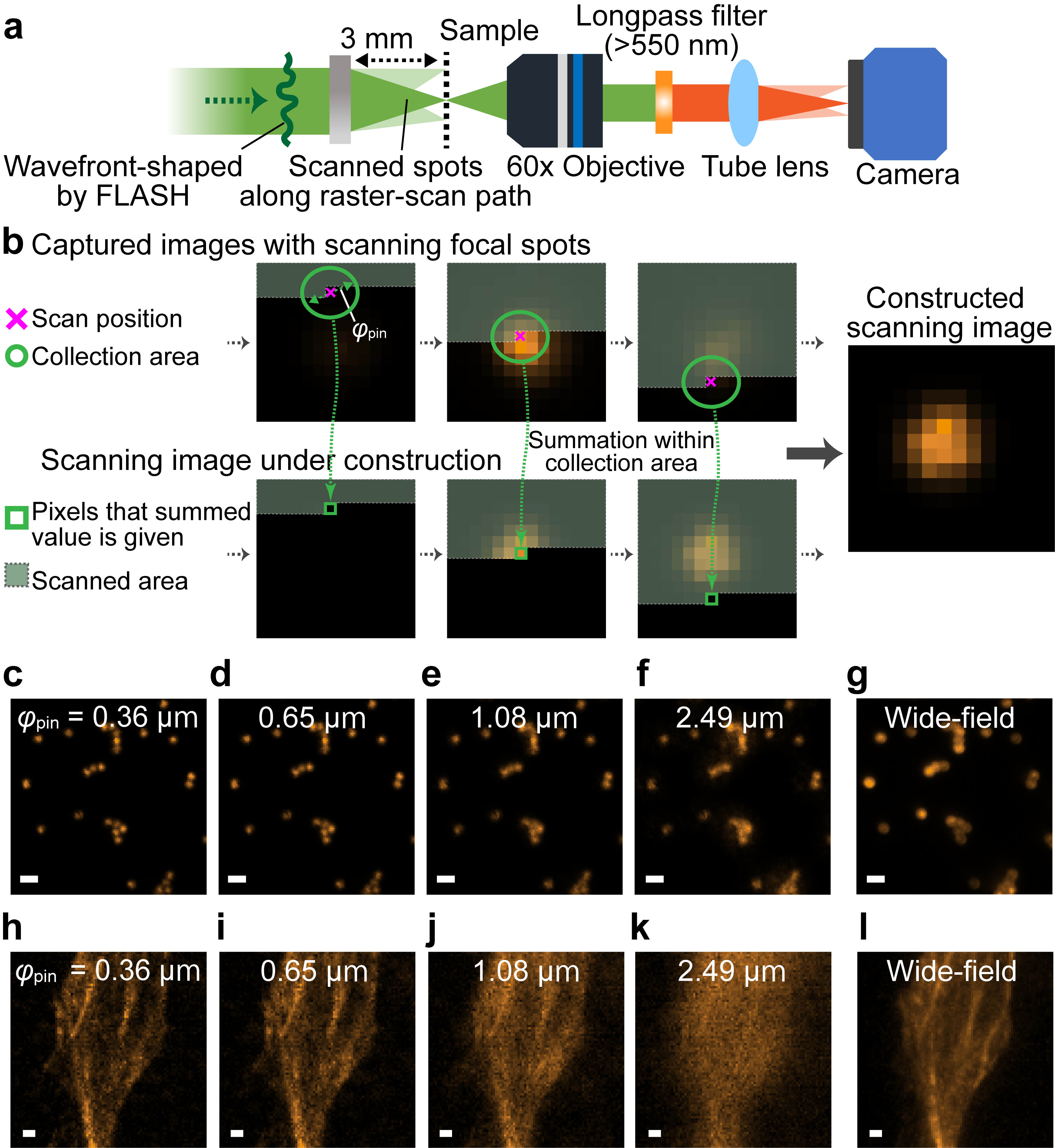
Supplementary Figure 12

**Supplementary Figure 12 |** Scanning fluorescence imaging with FLASH focusing. **a**, Optical setup for scanning imaging with a camera in transmission-mode. **b**, Schematic of a quasi-confocal detection method with the camera to construct a scanning image. **c-f**, Scanning fluorescence images of fluorescent microspheres with different collection diameters. **g**, Wide-field fluorescence image of the fluorescent microspheres. Scalebar: 1 μm. **h-k**, Scanning fluorescence images of fluorescence-stained HeLa cells with different collection diameters. **l**, Wide-field fluorescence image of the HeLa cells. Scalebar: 1 μm.

References

1. Jang, M. *et al.* Wavefront shaping with disorder-engineered metasurfaces. *Nat. Photon.* **12**, 84–90 (2018).

2. Boniface, A., Mounaix, M., Blochet, B., Piestun, R. & Gigan, S. Transmission-matrix-based point-spread-function engineering through a complex medium. *Optica* **4**, 54–59 (2017).

3. Vellekoop, I. M. & Aegerter, C. M. Scattered light fluorescence microscopy: imaging through turbid layers. *Opt. Lett.* **35**, 1245–1247 (2010).

4. Kakkava, E. *et al.* Selective femtosecond laser ablation via two-photon fluorescence imaging through a multimode fiber. *Biomed. Opt. Express* **10**, 423–433 (2019).

5. Čižmár, T., Mazilu, M. & Dholakia, K. In situ wavefront correction and its application to micromanipulation. *Nat. Photon.* **4**, 388–394 (2010).

6. Ruan, H. *et al.* Deep tissue optical focusing and optogenetic modulation with time-reversed ultrasonically encoded light. *Sci. Adv.* **3**, eaao5520 (2017).

7. Vellekoop, I. M., Cui, M. & Yang, C. Digital optical phase conjugation of fluorescence in turbid tissue. *Appl. Phys. Lett.* **101**, 081108 (2012).

8. Weiss, U. & Katz, O. Two-photon lensless micro-endoscopy with in-situ wavefront correction. *Opt. Express* **26**, 28808–28817 (2018).

9. Ji, N., Milkie, D. E. & Betzig, E. Adaptive optics via pupil segmentation for high-resolution imaging in biological tissues. *Nat. Methods* **7**, 141–147 (2010).

10. Reutsky-Gefen, I. *et al.* Holographic optogenetic stimulation of patterned neuronal activity for vision restoration. *Nat. Commun.* **4**, 1509 (2013).

11. Marshel, J. H. *et al.* Cortical layer–specific criticaldynamics triggering perception. *Science* **365**, eaaw5202 (2019).

12. Blochet, B., Bourdieu, L. & Gigan, S. Focusing light through dynamical samples using fast continuous wavefront optimization. *Opt. Lett.* **42**, 4994–4997 (2017).

13. Tzang, O. *et al.* Wavefront shaping in complex media with a 350 kHz modulator via a 1D-to-2D transform. *Nat. Photon.* **13**, 788–793 (2019).

14. Conkey, D. B., Caravaca-Aguirre, A. M. & Piestun, R. High-speed scattering medium characterization with application to focusing light through turbid media. *Opt. Express* **20**, 1733–1740 (2012).

15. Guo, Z. V., Hart, A. C. & Ramanathan, S. Optical interrogation of neural circuits in Caenorhabditis elegans. *Nat. Methods* **6**, 891–896 (2009).

16. Feldkhun, D., Tzang, O., Wagner, K. H. & Piestun, R. Focusing and scanning through scattering media in microseconds. *Optica* **6**, 72–75 (2019).

17. Panuski, C. L. *et al.* A full degree-of-freedom spatiotemporal light modulator. *Nat. Photon.* **16**, 834–842 (2022).

18. Smolyaninov, A., El Amili, A., Vallini, F., Pappert, S. & Fainman, Y. Programmable plasmonic phase modulation of free-space wavefronts at gigahertz rates. *Nat. Photon.* **13**, 431–435 (2019).

19. Benea-Chelmus, I. C. *et al.* Electro-optic spatial light modulator from an engineered organic layer. *Nat. Commun.* **12**, 5928 (2021).
